# Metabolic Vulnerability in the Neurodegenerative Disease Glaucoma

**DOI:** 10.3389/fnins.2017.00146

**Published:** 2017-03-30

**Authors:** Denise M. Inman, Mohammad Harun-Or-Rashid

**Affiliations:** Department of Pharmaceutical Sciences, Northeast Ohio Medical UniversityRootstown, OH, USA

**Keywords:** mitochondria, lactate, axonopathy, Wallerian degeneration, optic neuropathy

## Abstract

Axons can be several orders of magnitude longer than neural somas, presenting logistical difficulties in cargo trafficking and structural maintenance. Keeping the axon compartment well supplied with energy also presents a considerable challenge; even seemingly subtle modifications of metabolism can result in functional deficits and degeneration. Axons require a great deal of energy, up to 70% of all energy used by a neuron, just to maintain the resting membrane potential. Axonal energy, in the form of ATP, is generated primarily through oxidative phosphorylation in the mitochondria. In addition, glial cells contribute metabolic intermediates to axons at moments of high activity or according to need. Recent evidence suggests energy disruption is an early contributor to pathology in a wide variety of neurodegenerative disorders characterized by axonopathy. However, the degree to which the energy disruption is intrinsic to the axon vs. associated glia is not clear. This paper will review the role of energy availability and utilization in axon degeneration in glaucoma, a chronic axonopathy of the retinal projection.

## Metabolic vulnerability in glaucoma

Glaucoma is the leading cause of irreversible blindness worldwide (Tham et al., 2014). It blinds through the dysfunction and degeneration of retinal ganglion cell (RGC) axons that carry visual information from the eye to the brain (Calkins and Horner, 2012; Casson et al., 2012). There is emerging evidence for a critical role for energy management in the axon degeneration observed in this disease. In our work, we have been using the DBA/2J mouse model of glaucoma, an inbred strain that develops increased intraocular pressure (IOP) secondary to an iris pigment dispersion disease (John et al., 1998; Anderson et al., 2002). IOP is the main modifiable risk factor in primary open angle glaucoma, and lowering IOP is the mainstay of treatment. Increased IOP in the DBA/2J (D2) leads to optic neuropathy and eventual RGC death in a manner that emulates human patients with the most common forms of glaucoma, with pathology developing slowly over time (John et al., 1998), and occurring in contiguous retinal regions (Jakobs et al., 2005). IOP elevations in D2 mice are similar in scale to human patients, and the magnitude of these increases correlates with axon degeneration (Inman et al., 2006).

**Figure 1:**
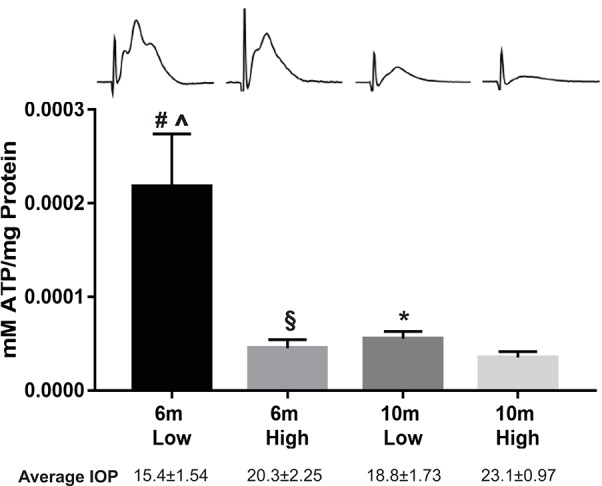
**Optic nerve ATP levels in the DBA/2J model of glaucoma at 6 and 10 months of age, separated into low and high intraocular pressure (IOP) groups**. Optic nerves were rapidly isolated then flash frozen in liquid nitrogen until assay. Upon thaw, the optic nerve was cut into small pieces while in 10% HClO_4_, then homogenized by sonication, and centrifuged at 4,500 rpm for 10 min at 4°C. The supernatant was collected, neutralized with 30 μl of 2.5 M KOH, then centrifuged at 14,000 rpm for 10 min at 4°C. The precipitate was removed, and supernatant kept on ice. Total ATP concentration was measured using the ATP bioluminescence assay kit (Roche), based on the ATP dependence of luciferase catalyzed oxidation of luciferin. Samples were diluted and mixed with the luciferase reagent and then absorbance was measured at 560 nm. Blank values were subtracted from the raw data, and ATP concentrations were calculated from a log–log plot of the standard curve data and normalized by the protein concentration. The values are expressed as millimoles of ATP per milligrams of protein. There was a significant difference in ATP levels in low vs. high IOP groups (*t*-test, #*p* = 0.012 for 6 m; ^*^p = 0.049 for 10 m), and a significant difference in the ATP levels across 6 m vs. 10 m at low (*t*-test, ∧*p* = 0.014) and high (*t*-test, §*p* = 0.028) IOP. Above: representative compound action potential traces from each group; below: average IOP for each group. Adapted from Baltan et al. (2010).

The potential role of energy availability and utilization in glaucoma became apparent through optic nerve physiology experiments. We measured compound action potential (CAP), the summation of all action potentials after stimulation, in freshly isolated D2 optic nerves. CAP amplitude decreased significantly by 6 months of age (Baltan et al., 2010), prior to measurable axon structural pathology (Inman et al., 2006) or axon transport deficit (Dengler-Crish et al., 2014) in this model. The reduction in elicited neural activity was inversely correlated with IOP elevation in the D2; the higher the IOP exposure within an age group, the lower the CAP amplitude (Baltan et al., 2010). Notably, there was no difference in CAP latency or duration in the D2 at 6 or 10 months, and no impact of the K^+^ channel blocker 4-aminopyridine. Both observations argue against significant changes in myelination as observed in multiple sclerosis-associated optic neuropathy in humans (Chan, 2002). However, when D2 optic nerves were subjected to oxygen-glucose deprivation and then allowed to recover, nerves from 6-month-old mice in the high IOP group demonstrated impaired recovery when compared to their low IOP companions. By 10 months of age, however, D2 optic nerves could not sustain recovery from oxygen-glucose deprivation in any mice, independent of IOP history. These results suggested that both IOP and age influence energy reserves in this pathology. To corroborate energy depletion, ATP measurements from D2 optic nerve showed significantly decreased ATP in the high IOP groups within each age, and significantly depleted ATP levels when comparing 6 to 10-month-olds within IOP group (see Figure 1 for summary) (Baltan et al., 2010). The low ATP and clear lack of energy reserve in the D2 optic nerve with increased IOP suggested glaucoma-associated metabolic dysfunction. Strikingly, the metabolic dysfunction in the axon occurs quite early in the D2, prior to overt structural changes in the optic nerve. This suggests that resolution of the metabolic dysfunction could prevent axon degeneration and preserve visual function.

The observations of a potential metabolic dysfunction in glaucoma raise a number of interesting possibilities about the mechanism of pathophysiology. Several fundamental concepts have implications for how glaucoma could be managed and what might be fruitful therapeutic approaches to combat the disease: (1) how energy is produced and utilized in axons, (2) how energy is associated with axon degeneration, (3) the nature of axon degeneration in glaucoma, and (4) how axon degeneration might be prevented. As discussed below, the entry points for the final destruction of the axon vary between neurodegenerative disorders but have much in common beyond initiation. The focus here is energy depletion, which arises from a number of circumstances, including ischemia, mitochondrial dysfunction, age-related changes in the NAD+-sirtuin-PGC-1α axis, loss of axon support factors, and activation of axon degeneration factors. Below we review the current understanding of energy production in white matter and possible intervention points in glaucoma and, by extension, other chronic axonopathies.

## Energy in axons

Energy is produced both within axons and around them using substrates delivered by the circulation and glial cells. Axons obtain most of their energy from ATP through oxidative phosphorylation in mitochondria. Glial cells that contact axons (oligodendrocytes and astrocytes) are capable of providing energy substrates to axons as well. This energy is necessary for axon function.

### Within axons: oxidative phosphorylation and glycolysis

The central nervous system prefers glucose as its substrate; glucose enters the brain (and retina and optic nerve) through glucose transporters. Neurons express GLUT3, a high-affinity glucose transporter (Maher et al., 1991), while astrocytes and endothelial cells express GLUT1 (Garcia-Caceres et al., 2016). Both GLUT3 and GLUT1 can be upregulated through insulin signaling (Simpson et al., 2008). Glucose is converted through glycolysis to pyruvate; the resultant pyruvate is fed into the Krebs cycle in mitochondria for the generation of ATP. Glucose in axons can also be used to produce reducing equivalents like NADPH via the pentose-phosphate pathway in order to maintain redox balance (Stincone et al., 2015). Neurons are highly oxidative and dependent on their mitochondria. ATP produced by mitochondria in the average ON axon can comfortably sustain the observed firing rate of action potentials, the maintenance of the resting potential, and maintenance processes, according to a computational model that drew on published values for rat CNS (Harris and Attwell, 2012). The model indicated mitochondria could support all optic nerve axons using roughly 50% of the ATP generation capacity. There is only a calculated ATP shortfall for small axons, those below 0.8 μm in diameter (Harris and Attwell, 2012). In humans, normal optic nerve axon mean diameter ranges from 0.72 ± 0.07 μm (Mikelberg et al., 1989), to 0.96 ± 0.07 μm (Quigley et al., 1988), and 1.00 ± 0.06 μm (Jonas et al., 1990). Even within these ranges, a significant portion of the axons in the human optic nerve are likely to experience ATP shortfall if the model accurately predicts energy availability. The same is true for mouse, with a frequency distribution of optic nerve fiber diameter that peaks at 0.7–0.9 μm; 50.4% of axons are 0.9 um in diameter or less (Honjin et al., 1977), suggesting that there could also be a mitochondrial-derived ATP shortfall in a large subset of axons. The energy sufficiency calculations also depend upon the axon having enough access to glucose.

**Figure 2:**
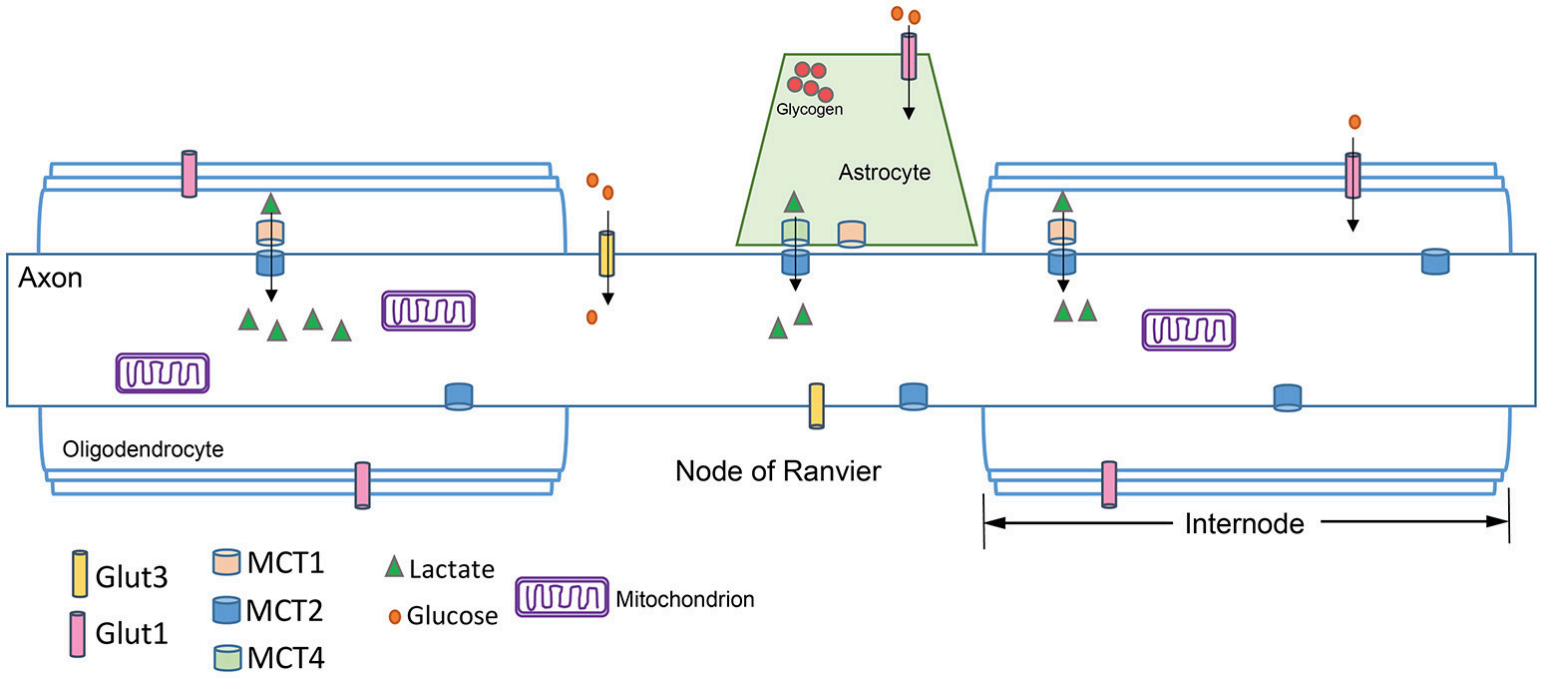
**Axon schematic showing the distribution of glucose transporters (GLUT), and monocarboxylate transporters (MCT) in the axon, astrocytes and oligodendrocytes**. GLUTs move glucose into the cell, while MCTs transport monocarboxylates such as lactate, pyruvate, and β-hydroxybutyrate. Glia express MCT1 and MCT4, while axons express MCT2. The kinetics of the MCTs are such that MCT1 is best suited for lactate export and MCT2 for lactate uptake because of its higher affinity for lactate (see text). The node of Ranvier is concentrated with ion channels (not shown) necessary for saltatory conduction. Astrocytes can interface with the axon at the node of Ranvier. Oligodendrocytes myelinate the axon; the region between nodes of Ranvier is the internode.

In general, there are adequate concentrations of GLUT3, the primary glucose transporter in neuronal membranes (Maher et al., 1991), to provide the necessary glucose levels to axons (Harris and Attwell, 2012). These estimates assume glucose diffusion to the middle of an internode quickly (see Figure 2); rat internodes average 240 μm (Ransom et al., 1991) while internode lengths in mouse ON average 110 μm (O'Meara et al., 2013). This internode length suggests glucose will readily diffuse; however, the predicted ATP shortfall in axons below 0.8 μm in diameter indicates a potential vulnerability of small axons. Small axons have light myelination, shorter internodes, slow conduction rates (Hursh, 1939), and more sparse mitochondria (Perge et al., 2009; Ohno et al., 2011). It has recently been demonstrated that glucose transporters are upregulated in response to activity in mouse myelinated axons (Saab et al., 2016), a potential solution to the possible lack of glucose in small axons. There are two caveats to this interpretation, however: The glucose transporters were upregulated in oligodendrocytes, not axons; and protein upregulation is not a dynamic response to momentary need. Axons likely rely on alternatives to glucose for maintenance of function.

### Around axons: glial-derived energy

Axons may be supplied with metabolic intermediates from astrocytes and oligodendrocytes to address potential shortfalls from glucose transport and diffusion. Several early studies demonstrated metabolic coupling between neuronal and glial compartments (Hamberger and Hyden, 1963; Pevzner, 1971, 1972). Glia-to-neuron energy substrate transfer was also revealed in early work on metabolic coupling between glia and neurons in the honeybee retina, indicating that glial cells released alanine that was taken up and metabolized by photoreceptor neurons (Tsacopoulos and Magistretti, 1996). In the mammalian CNS, glia mainly transfer monocarboxylates—L-lactate, pyruvate, or ketone bodies—rather than alanine, to neurons (Brown et al., 2003). The initial support for energy transfer from glia to axons came from studies of optic nerve explants that could maintain CAPs for approximately 30 min in the absence of glucose (Stys et al., 1991; Wender et al., 2000). Further study of optic nerve showed that CAP maintenance was dependent on transport of lactate (Wender et al., 2000; Tekkök et al., 2005). The critical evidence supporting intracellular metabolic substrate transfer is based on a number of observations, including: (1) the ability of glial cells to release L-lactate from their glycogen stores (Dringen et al., 1993), (2) the ability of neurons to take up L-lactate (Bouzier-Sore et al., 2003), (3) the cellular distribution of lactate dehydrogenase enzyme that converts lactate to pyruvate (Bittar et al., 1996), and (4) the cellular localization of monocarboxylate transporters (MCTs) within the CNS (Koehler-Stec et al., 1998; Pierre et al., 2002). Mounting evidence from different experimental studies indicates that oligodendrocytes and astrocytes significantly contribute to metabolic substrate transfer for axonal metabolic support.

**Figure 3:**
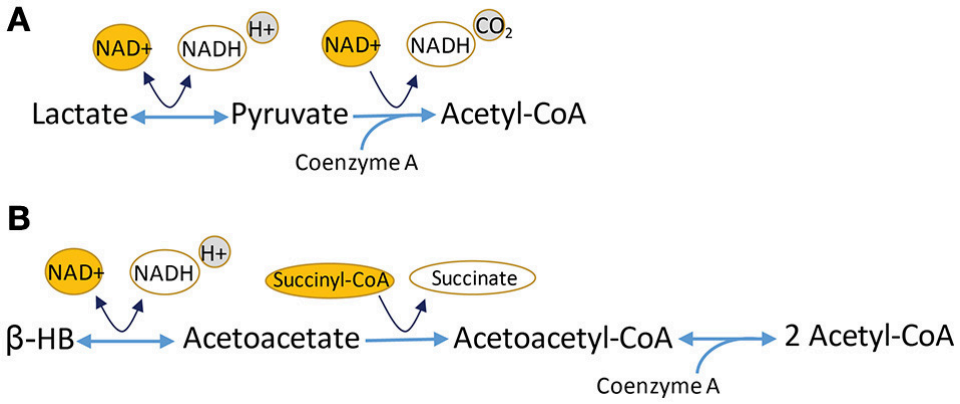
**Conversion of ketone bodies, including lactate and β-hydroxybutyrate (β-HB), intermediates that can be utilized in Krebs cycle. (A)** Lactate conversion to pyruvate is catalyzed by lactate dehydrogenase and requires NAD+. The reaction is reversible. Pyruvate is oxidized to acetyl-CoA, using NAD+ and giving off NADH and CO_2_. **(B)** β-HB can be utilized in Krebs cycle after a multi-step conversion reaction that results in the formation of two molecules of acetyl-CoA for every molecule of β-HB.

Lactate, a glycolytic end product, can be converted to pyruvate for utilization in the Krebs cycle (Figure 3). Evidence from many different experiments indicates that lactate is an efficient oxidative energy substrate for neurons (Pellerin et al., 1998). The conversion of lactate to pyruvate is catalyzed by lactate dehydrogenase (LDH), and requires NAD+ as a cofactor. Cells that produce lactate glycolytically, like astrocytes, are abundant in one LDH isozyme, while cells that use lactate as a substrate, including neurons, are enriched in another (Tsacopoulos and Magistretti, 1996). Lactate is transported between glia and neurons through the monocarboxylate transporters (MCTs). MCTs are bidirectional extracellular membrane channels that transport lactate, pyruvate, and ketone bodies across the membrane depending on their concentration gradient (Pierre and Pellerin, 2005). The MCTs work by first binding a proton then one molecule of lactate; the transporter undergoes a conformational change that releases the proton and the lactate on the other side of the membrane (Dubinsky and Racker, 1978). MCT isoforms differ in their substrate-binding affinities and kinetics, making MCT1 best suited for lactate export and MCT2 for lactate uptake. Perhaps unsurprisingly, MCT1 is expressed preferentially in oligodendrocytes and astrocytes (Lee et al., 2012), while MCT2 is expressed in neurons and axons (Pierre et al., 2002; Simpson et al., 2007), and MCT4 is expressed in astrocytes (Rafiki et al., 2003). MCT2 has a much greater affinity than MCT1 or MCT4 for lactate and pyruvate (Simpson et al., 2007).

### Glial glycolysis supports axons

Glial cells and neurons form a metabolic unit in the CNS, with glial cells providing energy substrates that ensure neural function. In *Drosophila*, neurons die when glial cell glycolysis is impaired, indicating that glycolysis is essential to neuron survival (Volkenhoff et al., 2015). The metabolic unit is supported by communication among cells; axon firing can lead to upregulation of glucose transporter-1 in oligodendrocytes in mouse (Saab et al., 2016). Once fortified with additional glucose, oligodendrocytes release lactate, thereby maintaining energy supplies to myelinated axons in the optic nerve. Moreover, evidence from multiple sclerosis patient tissue suggests that oligodendrocytes in low glucose conditions decrease their lactate release (Rone et al., 2016), possibly imperiling myelin maintenance but allowing cell survival. This suggests that glia are responsive to axon activity through greater release of metabolic substrate, but they have limits.

### Glycogen

Astrocytes have glycogen stores that are capable of being mobilized to provide glucose or lactate to axons (Pellerin et al., 1998; Erlichman et al., 2008). Mobilized glycogen allows for continued optic nerve CAP generation when mouse optic nerves are deprived of glucose (Wender et al., 2000). *In vitro* evidence suggests that lactate derived from glycogen breakdown is preferentially exported (Sickmann et al., 2005). The glycogen stores get broken down into lactate, which gets transferred to and used by axons, as shown by CAP failure when lactate transporters are blocked in periods of aglycemia in mouse optic nerve (Wender et al., 2000; Tekkök et al., 2005). Blocking glycogen breakdown accelerated CAP failure in optic nerve subjected to high-frequency stimulation (Brown et al., 2005). Furthermore, neuronal function can be maintained and neuronal death averted during hypoglycemia by increasing astrocytic glycogen stores in rat brain (Suh et al., 2007). These data demonstrate that lactate from glial glycogen stores can and will be used by axons under stress. In brain parenchyma, astrocytes have unique morphological structures and phenotypical features, which ideally position them to sense neuronal activity (Lundgaard et al., 2014) and respond with the suitable metabolic substrate to their surrounding microenvironment (Rouach et al., 2008). Is something similar at work in white matter, between astrocytes and axons? Gap junctions known to connect astrocytes and oligodendrocytes (Orthmann-Murphy et al., 2007) could facilitate movement of glucose or lactate from astrocyte to oligodendrocyte, thereby providing lactate or other substrates directly through the axon-facing MCT-1 on oligodendrocytes. Alternatively, astrocytes are known to contact nodes of Ranvier (Serwanski et al., 2017) and may deliver metabolic substrates directly.

### Energy and axon degeneration

Axon degeneration is a complex process that can initiate from different neuronal compartments, e.g., the soma, terminal boutons, or the axon itself. Regardless, the initial stages of neurodegeneration often manifest in the axon compartment. Axon degeneration mechanisms can share three common elements: intra-axonal Ca^++^ dysregulation, axon transport deficits, and mitochondrial dysfunction (Fagiolini et al., 1997; Stys, 2004). Both axon transport deficits and mitochondrial dysfunction are energy-related pathologies. For example, molecular motors require energy for axon transport. Earlier work on this subject posited that mitochondrial dysfunction would preclude axon transport until it was demonstrated that molecular motors can use extra-mitochondrial sources of energy (Zala et al., 2013). This may dissociate axon transport deficit from mitochondria with regards to ATP demand; however, pathological change in mitochondrial dynamics can alter transport through other mechanisms (Crish and Calkins, 2011; Misko et al., 2012). Metabolic dysfunction could contribute to axon transport deficit, independent of mitochondria, through lack of substrate delivery to the axon. Anterograde axon transport deficit is known to be an important alteration in the pathology of glaucoma (Crish et al., 2010; Dengler-Crish et al., 2014). As will be discussed below, axon transport is required to maintain axon structure and function as well as traffic survival factors for the neuron (see Vrabec and Levin, 2007; Milde et al., 2013).

### Mitochondria and degeneration

Investigation into the mechanisms of axon degeneration underscores the central role of energy availability and mitochondria in keeping the axon compartment alive. For example, injured axons in zebrafish rapidly degenerate without mitochondria (Campbell et al., 2014). In *C. elegans*, mitochondrial targeting to axons can protect them from degeneration (Rawson et al., 2014). Similarly, releasing mitochondria from their syntaphilin tethers in axotomizedcultured cortical mouse axons can rescue energy deficits (Zhou et al., 2016), demonstrating the importance of ATP homeostasis to axon survival after injury. Some have determined mitochondria to be the site of axon protection (Avery et al., 2012), though preventing mitochondrial transport into axons prior to injury slowed, but did not stop, the course of axon degeneration in a *Drosophila* axotomy model (Kitay et al., 2013). Depolarizing mitochondria does not increase the rate of Wallerian degeneration in superior cervical ganglion axons *in vitro*, indicating that degeneration-associated Ca^++^ increases are extra-mitochondrial (Loreto et al., 2015). Axon regeneration, quite separate from degeneration, in mouse RGC axons (Cartoni et al., 2016) and *C. elegans* nerve cord axons (Han et al., 2016), does require mitochondria.

### Glial lactate critical for axon survival

Recent experimental data demonstrates that MCTs are critical for maintaining axon health and integrity. Mice with 50% lower than normal MCT1 expression developed axonopathy in the optic nerve, suggesting that axons are quite sensitive to reduction in the provision of lactate from oligodendrocytes (Lee et al., 2012). Neurons in organotypic cultures could not survive without MCT1, and axons without it degenerated. MCT1 was also shown to be reduced in patients with amyotrophic lateral sclerosis (ALS) and in a mouse model of ALS (Lee et al., 2012). Deletion of MCT1 in oligodendrocytes substantially reduces the availability of local energy metabolites to the axon (Lee et al., 2012), potentially affecting the axonal energy-dependent processes such as axonal transport (Nave, 2010).

The crucial role of MCT1, a mover of energy substrate from the oligodendroglial compartment to axons, in axon function and survival spurred investigation into the regulation of energy exchange between oligodendrocyte and neuron (Saab et al., 2016). Stimulation of oligodendroglial NMDA receptors in response to axonal glutamate release upregulates glucose transporter GLUT1 in the oligodendrocyte. Increased uptake of glucose in the myelin as a result of GLUT1 expression ensures greater glucose delivery to the oligodendrocytes at a time when the axon is engaged in high spiking activity, thereby establishing a path to greater lactate release by the oligodendrocyte to the myelinated axon. Axons recovered from oxygen-glucose deprivation better with lactate than with glucose, indicating the oligodendrocytes were not merely passing along glucose to the axons (Saab et al., 2016).

A multi-pronged approach to supplying axons with energy, as outlined in the section on energy described above, is necessary because of the dire consequences of axonal energy depletion. Axons expend the bulk of their energy budget on the Na^+^-K^+^ ATPase which moves these ions across the axolemma during and after action potential firing (Ritchie, 1967). Without sufficient energy for Na^+^-K^+^ ATPase function, axons experience Na^+^ and Ca^++^ overload and then structural decline when Ca^++^-dependent proteases such as calpain (and potentially others) lay waste to the cytoskeleton, disrupting axon transport, physiology, and other vital axon maintenance processes. Extracellular Ca^++^ influx is both necessary and sufficient for axon degeneration (Court and Coleman, 2012); it is part of a “final common pathway” for various mechanisms of axon degeneration such as developmental axon pruning and Wallerian degeneration (Yang et al., 2013); reviewed in (Stirling and Stys, 2010; Tsutsui and Stys, 2013; Conforti et al., 2014).

### Energy and mechanisms of axon degeneration: wallerian

**Figure 4:**
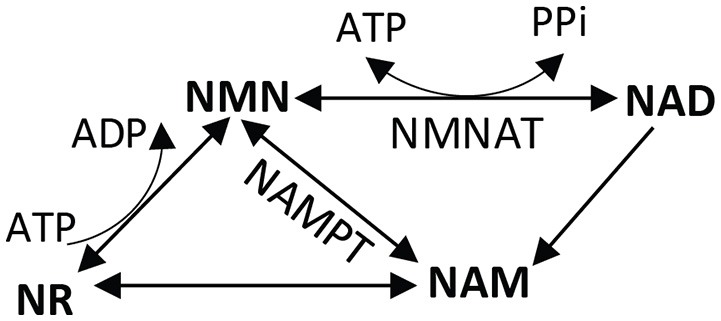
**Nicotinamide dinucleotide (NAD+) salvage pathway**. NAD+ can be synthesized *de novo* using tryptophan (not shown), or through a salvage pathway using vitamin precursors niacin (vitamin B3), nicotinamide (NAM), or nicotinamide riboside (NR). In the salvage pathway, NMNAT catalyzes the formation of NAD+ from NMN and ATP. The reverse reaction converts NAD+ into NMN, giving off ATP. NMN, nicotinamide mononucleotide; NAD, nicotinamide dinucleotide; NMNAT, nicotinamide mononucleotide adenylyltransferase; NAMPT, nicotinamide phosphoribosyltransferase.

A role for energy in the mechanism of axon degeneration emerged from research into the mechanism of axon protection in the Wallerian degeneration-slow (Wld^s^) mouse. Wld^s^ mice carry a naturally occurring mutation that greatly slows axon degeneration, allowing axons that are separated from the cell body to maintain structural and conduction integrity for 2 weeks after transection in mice (Lunn et al., 1989). In a series of follow-up studies, it was demonstrated that the slow degeneration phenotype was intrinsic to the axon (Perry et al., 1990a) and was controlled by a single autosomal dominant gene (Perry et al., 1990b; Lyon et al., 1993). It was ultimately determined that the Wld^s^ gene encodes a chimeric protein, a fusion of the N-terminal 70 amino acids of ubiquitination factor E4B (Ube4b) with nicotinamide mononucleotide adenylyltransferase-1 (NMNAT1) (Conforti et al., 2000; Mack et al., 2001). Subsequent research activity has indicated that the NMNAT1 portion of the fusion protein is primarily responsible for the Wld^s^ phenotype, as shown in in dorsal root ganglion culture (Araki et al., 2004). It is the ATP-dependent activity of NMNAT that converts nicotinamide mononucleotide (NMN) to NAD+ (Figure 4). NAD+ is an essential coenzyme in four steps of the Krebs cycle, an essential cofactor in the GAPDH step of glycolysis, and the conversion of lactate into pyruvate requires NAD+, reducing it to NADH. Determining that NMNAT activity contributes to axon survival after axotomy placed energy as a central issue to the mechanism of axon degeneration.

Given the critical role of NMNAT in axon survival after injury, logic would dictate that the product of NMNAT activity, NAD+, is the currency of axon protection. Mammals use a variant of vitamin B3, nicotinamide, as the precursor for NAD+ synthesis (Galli et al., 2013) (Figure 4). Roughly 50% of the pyridine nucleotide NAD+ pool is in mitochondria, and 50% is in the cytoplasm in cultured neurons (Alano et al., 2010). NADH and NAD+ cannot themselves be transported into or out of the mitochondria. Cytosolic NAD+ depletion has been shown to block glucose utilization, consistent with its requirement in glycolysis. NAD+ is regenerated in cells by the activity of lactate dehydrogenase, through the electron transport chain in mitochondria, or the movement of malate and/or aspartate transport across the inner mitochondrial membrane. The major consumers of NAD+ in a cell include poly (ADP-ribose) polymerases (PARPs), and sirtuins (class III histone deacetylases) (Blander and Guarente, 2004; Belenky et al., 2007). Strikingly, eliminating two major NAD+ consuming enzymes (PARP1 and CD38) does not prevent axon degeneration in mouse sciatic nerve transection (Sasaki et al., 2009). *In vivo*, NMNAT1 overexpression was not sufficient for axon protection, but NMNAT3 targeted to the mitochondrial matrix protected sciatic nerves after axotomy (Yahata et al., 2009). NMNAT3 likely regenerates NAD+ in the mitochondria for cellular energetics (Lau et al., 2009). Since the mitochondrial NAD+ pool is segregated from the cytoplasmic, NMNAT3-based protection from degeneration implicates mitochondrial dysfunction in the energy depletion that contributes to axon degeneration. The mitochondria isolated from these axons had normal respiratory chain components but were making more ATP (Yahata et al., 2009). The centrality of NAD+ in energy production and utilization suggest a mechanism linking loss of mitochondrial function to axon degeneration (Di Lisa and Ziegler, 2001).

Mitochondria isolated from Wld^s^ -expressing, protected mouse axons exhibit increased ATP production (Yahata et al., 2009) and enhanced Ca^++^ buffering (Avery et al., 2012). Importantly, Wld^s^ can ameliorate decreases in ATP after axon injury *in vitro* (Wang et al., 2005). There are multiple NMNATs, each one localized to a cellular compartment. NMNAT1 is nuclear, NMNAT2 is cytoplasmic and associated with Golgi-derived vesicles, and NMNAT3 is found in the mitochondria (Ali et al., 2013). Each of these NAD+-synthesizing enzymes has been investigated individually and found to impact axon survival. Mutations in NMNAT1 cause Leber's congenital amaurosis, a retinal degeneration disease (Falk et al., 2012). Targeting NMNAT1 expression to the cytoplasm then inducing ocular hypertension protects RGCs from death (Zhu et al., 2013). The protective effect of a cytoplasmic version of NMNAT1 depended upon mitochondrial axon transport (Fang et al., 2014a). NMNAT2, however, has turned out to be the essential axon protection factor; when not transported to the axon, a degenerative program ensues (Gilley and Coleman, 2010). Mutations that extend the half-life of NMNAT2 increase its ability to delay axon degeneration, beyond that observed with the Wld^s^ mutation (Milde et al., 2013). The Wld^s^ protein can protect a cut axon for up to 4 h *in vitro*, while NMNAT2 depletion within 5 h commits the axon to degeneration (Gilley and Coleman, 2010; Wang et al., 2015). The longer half-life of Wld^s^ over NMNAT2 may explain its ability to radically extend axon survival.

**Figure 5:**
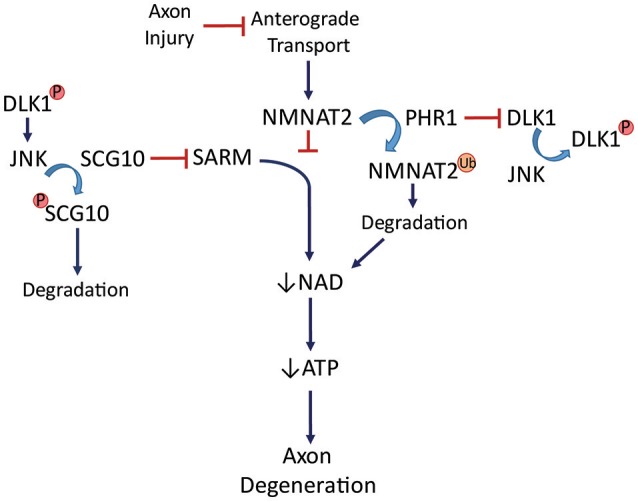
**NMNAT2 is transported into the axon to support/protect axon integrity**. PHR1, an E3 ubiquitin ligase, can ubiquitinate NMNAT2, promoting its degradation. This leads to decreases in NAD+, low ATP levels, and eventual axon degeneration. SARM activation also leads to axon degeneration through loss of NAD+ and ATP. Ubiquitination-deubiquitination of PHR1 keeps DLK1 in balance until it is phosphorylated by JNK. DLK1 can then activate JNK, which phosphorylates SCG10, leading to its degradation. Axonal degeneration can be blocked by SCG10. Loss of SARM or PHR1 delays degeneration. See text for details.

Identification of NMNAT2 as an axon protection factor has been accompanied by identification of two proteins that appear to be axon death factors (see Figure 5). PHR1, an E3 ubiquitin ligase, promotes axon degeneration after injury (Babetto et al., 2013) by influencing the turnover of NMNAT2. PHR1 deletion delays Wallerian degeneration. SARM1, a Toll-like receptor adaptor protein tethered to the mitochondria (Kim et al., 2007; Summers et al., 2014), contributes to axon degeneration such that its deletion significantly delays Wallerian degeneration after nerve transection (Osterloh et al., 2012). The mechanism of that delay includes increased NAD+ synthesis in the axon (Gerdts et al., 2015). SARM1 activation rapidly breaks down NAD+ after injury. SARM1 is required for activation of a post-injury MAPK cascade that disrupts energy homeostasis through ATP depletion in the axon (Yang et al., 2015). Cut neurites from SARM1−/− mice showed higher extracellular acidification rate (glycolysis) and higher maximal respiration than wildtype (Godzik and Coleman, 2015), suggesting a complex picture of the role of oxidative phosphorylation and glycolysis in maintaining ATP levels in injured axons. Isolated neurites show just a partial view of what is likely happening *in vivo*, where oligodendrocytes and astrocytes are capable of contributing to the overall energy scheme.

The prospect of axon survival has become quite complex with the further identification of elements capable of contributing to axon degeneration. Dual leucine zipper kinase (DLK), a MAP3K upstream of JNK, is necessary and sufficient for RGC death after optic nerve crush (Welsbie et al., 2013). Inhibiting the JNK cascade in DLK1−/− sensory neuron cultures delays Wallerian degeneration (Miller et al., 2009), suggesting that DLK1 acts through JNK to promote axon degeneration. There is some disagreement regarding the role of DLK1 in RGCs. Axon degeneration was not delayed after optic nerve crush in DLK1-deficient mice (Fernandes et al., 2014). While this suggests that kinases other than DLK1 may be capable of JNK activation for axon degeneration, a separate study showed that phosphorylation of c-Jun, a target of JNK, was dependent on DLK1 after optic nerve crush (Watkins et al., 2013). DLK1 was significantly upregulated in post-crush optic nerve and governed both the pro-apoptotic and pro-regenerative responses of RGCs (Watkins et al., 2013). JNK works in the commitment phase of the degeneration cascade since inhibiting it 3 h after axotomy of mouse or *Drosophila* axons was not sufficient to prevent axon loss (Miller et al., 2009). A protein phosphorylated early after axotomy in the distal segments of sensory nerve, SCG10, is a microtubule binding protein and JNK substrate whose phosphorylation targets it for degradation. SCG10 can delay degeneration of crushed mouse optic nerve if overexpressed (Shin et al., 2012). Loss of SCG10 alone, however, does not lead to axon degeneration. JNK regulates SCG10 turnover, linking it to DLK1. Interestingly, SCG10 is lost after axotomy even when NMNAT overexpression prevents axon degeneration (Shin et al., 2012).

After axotomy, PHR1 contributes to NMNAT2 degradation while SARM1 is similarly contributing to NAD+ depletion (Figure 5). DLK1 activates JNK, which ensures loss of SCG10. These events commit an axon to the degeneration cascade, and it is not yet known how the various elements interact or the precise timing. There are most certainly modifiers of the cascade as well as variations by mode of injury and type of neuron, as will be discussed below. Caveats to the general degeneration scheme include the fact that some of these mechanisms have been worked out *in vitro*, in isolated neurites. While this has established that Wld^s^, SARM, and DLK-1 are cell-autonomous in their impact on axon degeneration, the various unexplained aspects of degeneration mechanisms are being assiduously investigated.

### Glaucomatous axonopathy: what form does it take?

**Figure 6:**
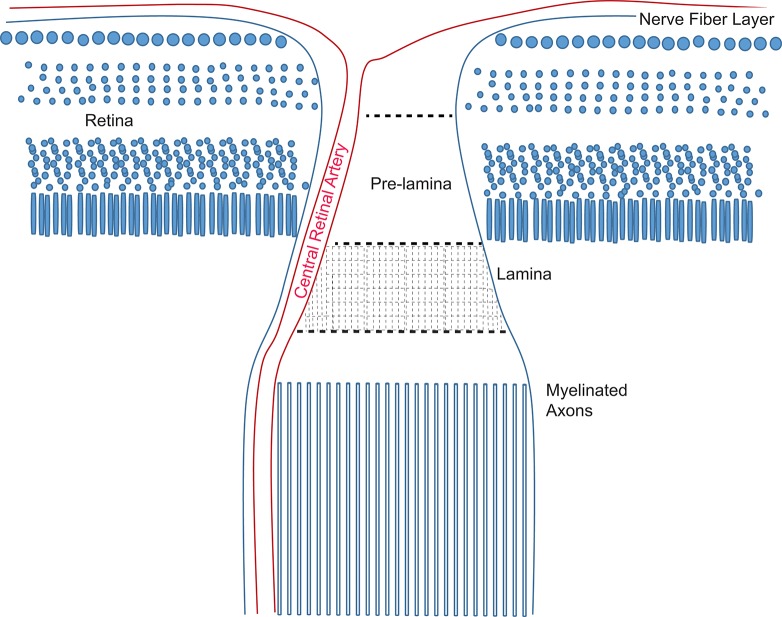
**Schematic of the optic nerve head in the mouse**. Retinal ganglion cell axons converge at the optic disk and then make a ~90° turn to form the optic nerve. The pre-lamina region is formed from the fasciculated axons as they traverse the retina. Columns of astrocytes maintain the fasciculated bundles of unmyelinated axons through the lamina. Beyond the lamina, retinal ganglion axons are myelinated by oligodendrocytes.

The success of axon protection strategies that have emerged from investigation into the Wld^s^ mutant applied to glaucoma may enable a determination about the mechanisms of axon degeneration in this disorder. In Wallerian degeneration, axons degenerate caudal to a lesion and in an asynchronous way over a population of axons within a nerve (Waller, 1850). The nature of the injury dictates its course since Wld^s^ axons degenerate anterogradely (from the injury toward the synapse) after transection but retrogradely (from the synapse back toward the injury) after crush injury in peripheral nerve (Beirowski et al., 2005). Wallerian degeneration can be triggered even without physical axonal injury (Ferri et al., 2003; Gilley and Coleman, 2010). In distal axonopathy, for which there is evidence in mouse models of glaucoma (Schlamp et al., 2006; Crish et al., 2010), an axon degenerates from the distal-most point and moves retrogradely. It may be the case that “dying back,” or distal axonopathy is not separable from Wallerian degeneration in glaucoma, especially if glaucoma injury resembles a crush injury with retrograde degeneration. Support for shared mechanisms of distal axonopathy and Wallerian degeneration comes from models of Charcot-Marie-Tooth disease, a distal axonopathy that is significantly slowed by the expression of the Wld^s^ (Meyer zu Horste et al., 2011). Wld^s^ can also reduce the number of axon varicosities, enlarged portions of axons containing protein aggregates that are hallmarks of most neurodegenerative diseases. Despite this, Wld^s^ cannot delay all axon degeneration (Vande Velde et al., 2004), and there may be age-related limits to axon degeneration delay (Samsam et al., 2003). Wld^s^ has been shown to effectively delay the optic neuropathy of glaucoma in the mouse (Howell et al., 2007; Beirowski et al., 2008), though it did not prevent or delay RGC death. A comparison of pre-laminar and laminar axons (see Figure 6) in the optic nerve head of D2 mice with glaucoma show a significant decrease in axon number from pre-lamina to lamina, suggesting that axon damage initiates in the pre-lamina and distal axons degenerate (Howell et al., 2007). This would be consistent with an anterograde Wallerian degeneration as observed in transected axons (Beirowski et al., 2005). However, axon endbulbs can be observed throughout the optic nerve in the D2 mouse model of glaucoma (Buckingham et al., 2008; Crish et al., 2010; Dengler-Crish et al., 2014), suggesting a retrograde degeneration course that would be more in alignment with the response to crush injury (Beirowski et al., 2005). Consistent with this, one analysis of D2 optic nerve showed a distal to proximal pattern of degeneration that preceded loss of the corresponding RGCs (Schlamp et al., 2006). Without the ability to monitor the axons along their course, it cannot be ruled out that the end bulbs and axon fragmentation observed beyond the optic nerve head are distal portions of axons with an initiating injury at the optic nerve head.

Neurotrophin deprivation has been considered an important potential mechanism of RGC decline and death. Manipulating receptor tyrosine kinase (Trk) signaling concomitant with growth factor provision has proven to be supportive of RGC survival (Cheng et al., 2002; Lebrun-Julien et al., 2009; Weber and Harman, 2013). Developmental axon pruning is an example of axon degeneration that can be triggered by loss of growth factor support from target areas. Axon pruning may share downstream mechanisms of degeneration with post-injury Wallerian degeneration, but the commitment phase differs. Wld^s^ cannot prevent pruning in mice or *Drosophila* (Hoopfer et al., 2006). Since Wld^s^ mice show normal CNS circuitry instead of a gross overabundance of unpruned connections, this is not unexpected. Evidence indicates there may be two mechanisms by which axons get pruned, one that is caspase-dependent and the other requiring loss of NAD+; the two may work together or run parallel to eliminate unneeded circuitry (Schoenmann et al., 2010). Growth factor deprivation initiates activation of caspases; Wld^s^ and cytoplasmic NMNAT1, even SARM1 deletion (Gerdts et al., 2013), can protect against nerve growth factor (NGF) withdrawal-induced axon degeneration. DLK-1, after trophic deprivation, is JNK dependent and contributes to both axon degeneration and cell body apoptosis (Ghosh et al., 2011). Interestingly, DLK1 knockout in a optic nerve crush model did not protect RGC axon structure or function as measured by CAP amplitude (Fernandes et al., 2014), suggesting that degeneration in these axons is governed differently than those in development (Hoopfer et al., 2006) and potentially other sensory systems (Miller et al., 2009). Others determined that deficiency in DLK1 protected RGC somas from apoptosis and proximal axons from degeneration after optic nerve crush (Watkins et al., 2013). The pro-apoptotic factor BAX, when deleted in mice of the D2 background, preserved RGC somas but axons degenerated (Libby et al., 2005). These data indicate that degeneration of the optic nerve in the D2 model of glaucoma may be caspase-independent. The preponderance of evidence suggests that glaucomatous degeneration occurs through Wallerian degeneration rather than trophic deprivation-related pathways, though the initial kinase signaling has yet to be determined.

## Energy and glaucoma-specific axon degeneration

### RGCs vulnerable to energy depletion

RGCs are not uniformly susceptible to cell death in glaucoma (Li et al., 2006; Della Santina et al., 2013), and whether specific axons are particularly vulnerable to degeneration is not known. In the cat optic nerve, 50% of the axons are in the category of small and lightly myelinated, and are therefore potentially susceptible to ATP shortfall. It is believed these axons correspond to γRGCs (Williams and Chalupa, 1983) which are the W cells by functional type. W cells include on- or off-tonic, on- or off-phasic, and on-off phasic cells (Fukuda et al., 1984; Watanabe et al., 1993). Axon caliber correlates relatively well with soma size (Huxlin and Goodchild, 1997; Coombs et al., 2006), suggesting that the sizable group comprising the slowest conducting axons should include RGCs with small to medium cell somas. A number of studies have determined that OFF transient RGCs show early morphological and functional changes in the microbead injection and laser photocoagulation models of glaucoma (Della Santina et al., 2013; El-Danaf and Huberman, 2015; Ou et al., 2016). These OFF transients are believed to be αRGCs, those with large somas and extensive dendritic arbors; however, there are a number of RGC morphological types with arbor stratification at 50% of inner plexiform layer (IPL) depth typical of OFF transient cells (Della Santina and Ou, 2016) that could have small to medium cell somas and axons possibly in the smaller ranges. The D2 CAP data (Figure 1) and OFF transient data are not necessarily in opposition since the traces clearly show that CAPs are lost from all axon calibers (Baltan et al., 2010); large αRGCs can be preferentially lost among other cells that also succumb. Interestingly, after optic nerve transection in mouse, αRGCs preferentially survive, including OFF transients; when treated with osteopontin and IGF-1 or by downregulating PTEN, regeneration only occurs in αRGCs (Duan et al., 2015). Though αRGCs are a diverse group, it would seem unlikely that cells particularly susceptible to raised IOP would be resistant to axon transection.

### Mitochondria in glaucoma

To understand the mechanism of metabolic dysfunction in D2 optic nerve, the mitochondria would be a reasonable starting point for investigation since axons obtain most of their energy from ATP through oxidative phosphorylation. One very relevant study determined that mitochondria isolated from patients resistant to optic neuropathy—exposed to high IOP but absent pathology—showed greater levels of systemic “mitochondrial efficiency,” including higher rates of ADP phosphorylation, hyperpolarized mitochondrial membrane potential, and enhanced Ca^++^ buffering capacity compared to control and glaucoma patient groups (Lascaratos et al., 2015). These observations single out the mitochondrial function as a potential biomarker for individuals susceptible to increased IOP. Our electron microscopic analysis of mitochondria in the D2 optic nerve is consistent with lower mitochondrial support in glaucoma patients. We observed a linear relationship between mitochondrial volume and axon volume in DBA/2J-Gpnmb^+^ (control strain that does not develop glaucoma) and pre-pathological D2 optic nerve. That relationship is abolished in the transport dysfunctional D2 optic nerve, for which we observed axon volumes that were not matched by appropriate mitochondrial volumes (Kleesattel et al., 2015). Interestingly, CAP amplitude measures suggest that the third peak of the CAP trace, corresponding to the slowest conducting axons, are lost earliest and in the greatest numbers, and in accordance to magnitude of IOP exposure, in the D2 mouse model of glaucoma (see Figure 1; Baltan et al., 2010).

What ties metabolism to susceptible RGCs? In short, very little is directly known about the specifics of OFF transient cell energy homeostasis, though mitochondrial distribution could be a factor. Whereas mitochondria take up greater axon volume in unmyelinated axons, lightly myelinated axons in mouse (Ohno et al., 2011) and guinea pig have slightly fewer mitochondria than predicted (Perge et al., 2009). Mitochondria in the D2 optic nerve are significantly smaller and possess reduced cristae with aging and increased IOP (Coughlin et al., 2015); this supports widespread fission of these organelles, as has been observed by others (Ju et al., 2008). Smaller mitochondria with reduced cristae have less machinery for oxidative phosphorylation, and therefore, lower energetic capacity. This would spell particular trouble if the optic nerve were ever to experience glucose shortage (for example, if glucose transporters were downregulated) because functional mitochondria are required for an axon to survive on lactate. Lactate conversion to pyruvate bypasses glycolysis. The resultant pyruvate is converted to acetyl-CoA for use in Krebs cycle (Figure 3). The intermediates produced there are used to establish the electron transport chain and the proton motive force for ATP Synthase (Complex V) and ATP production. Hence, survival on lactate requires functional mitochondria.

Unfortunately for axon survival, poorly functioning mitochondria are not being efficiently recycled in the D2 optic nerve (Coughlin et al., 2015). By not being replaced, these mitochondria can contribute to metabolic vulnerability by producing lower levels of ATP and comparatively more reactive oxygen species (ROS). Mitochondrial biosynthetic proteins such as PGC-1α decrease with age, including in the retina of D2 mice (Guo et al., 2014). Low PGC-1α levels likely limit mitochondrial biogenesis. With no prospect of generating new mitochondria, the lack of recycling of malfunctioning organelles is likely a survival mechanism.

Investigating the impact of upregulating mitophagy in glaucoma could resolve whether maintaining compromised mitochondria can ensure continued, albeit weakened, axon function. Some insight can be gleaned from a study in which autophagy was inhibited in glaucoma using 3-methyladenine (Seglen and Gordon, 1982). The authors observed significant axon degeneration after inhibiting autophagy, and axon protection when promoting autophagy through rapamycin treatment (Kitaoka et al., 2013). Conflicting results have accompanied those examining the role of autophagy in glaucoma, with autophagy induction detrimental to RGC soma survival in an ocular hypertension glaucoma model (Park et al., 2012), but rapamycin-induced autophagy protective of RGC somas after axotomy (Rodriguez-Muela and Boya, 2012).

### Glial cells

As outlined above, astrocytes and oligodendrocytes can exert significant control over the availability and utilization of energy in the optic nerve. Analyses of optic nerve head and axonal glia in glaucoma to date have focused on morphological change (Lampert et al., 1968; Dai et al., 2012; Sun and Jakobs, 2012; Lye-Barthel et al., 2013; Bosco et al., 2016; Cooper et al., 2016), or cell loss. In the D2 optic nerve, astrocyte hypertrophy occurred early in the disease process and was maintained; oligodendrocytes were lost only after axon loss (Son et al., 2010). Alternatively, a model of laser induced ocular hypertension led to significant oligodendrocyte loss within 1 week of IOP elevation (Nakazawa et al., 2006). Oligodendrocyte loss could be ameliorated with an antibody against TNFα, suggesting a role for microglial-associated inflammation in the loss of myelin. Specific attributes of oligodendrocytes in glaucoma have not been investigated, though NG2+ cells proliferate and generate new oligodendrocytes after axon degeneration in the optic nerve (Son et al., 2010). In considering what is understood about the other macroglial cell in the optic nerve during glaucoma, the relationship between astrocyte hypertrophy and metabolic support of axons is unknown. These knowledge gaps provide many fertile areas of investigation for the metabolic role of optic nerve glia in glaucomatous neurodegeneration.

## Addressing energy failure in glaucoma

Glaucoma is an asynchronous degeneration of the optic nerve. Does axon degeneration occur from loss of protection factors or activation of death factors, or is it both? Where and when does energy dysregulation occur? How does one support axons before they fail? One approach to prevent axon degeneration is to develop ways to target cells that are likely to succumb. As noted above, specific RGCs and compartments seem particularly susceptible. As research refines the susceptible populations, targeting strategies can also become more sophisticated, though certain protection strategies may show positive consequences to targeting all RGCs. Aging remains an important risk factor, as suggested by the significant decreases in ATP with age (Figure 1) as well as degree of IOP exposure. Various energy-related approaches have been used to prevent axon degeneration in models of neurodegeneration, including providing creatine, manipulating members of the NAD+-sirtuin-PGC1α axis, and upregulating the NMNATs. Strategies used to protect against axon degeneration in glaucoma in particular consist of providing glucose, upregulating NMNAT1 and NMNAT3, providing ketone bodies, and inhibiting histone deacetylases (HDACs).

### Glucose

Protection from glaucoma-induced optic neuropathy can occur by increasing energy substrates such as glucose. In a rat laser photocoagulation model of glaucoma, induction of hyperglycemia 4 days prior to ocular hypertension was shown to decrease axon loss in the optic nerve by more than 50% and increase ganglion cell survival also by roughly 50% (Ebneter et al., 2011). While glial activation was decreased in the optic nerve, retinal glial showed comparable levels of reactivity (microglia and astrocytes/Müller glia). A caveat to this study included the relatively brief study period (2 weeks) (Ebneter et al., 2011). Longer periods of hyperglycemia (6 weeks to 3 months) after ocular hypertension did not show neuroprotection in the retina (Kanamori et al., 2004a), though a distinction between neuronal and glial cell death was not made. A critical issue with increasing blood glucose levels is that hyperglycemia does not necessarily translate into greater glucose availability to the CNS without matching levels of high affinity glucose transporters such as GLUT3, or GLUT1. Whereas GLUT1 appears to be upregulated by NMDA-R activation (Saab et al., 2016), insulin can promote translocation of GLUT1 and GLUT3 to the cell surface (Uemura and Greenlee, 2006). In chronic glaucoma models, the likelihood of NMDA-R activation sufficient to upregulate GLUT1 seems unlikely because RGCs generally fire at low rates (more than 90% fire at 10 Hz or less) (Koch et al., 2004), and axons are being lost. The ectopic glutamate release observed in the optic nerve head in one glaucoma model (Fu and Sretavan, 2012) would not be directed at oligodendrocytes because axons in the nerve head are unmyelinated, but might its target be laminar astrocytes in an attempt to upregulate glucose transporters? Episcleral cauterization to initiate ocular hypertension in rats led to increased insulin and insulin-like growth factor receptor levels, concomitant with phosphorylated Akt, in retina in the acute post-injury period (Kanamori et al., 2004b). In the R28 rat retina neural cell line subjected to serum deprivation, insulin has been shown to be a retinal neuron survival factor by acting through the PI3K-Akt signaling pathway to decrease caspase-3 activation (Barber et al., 2001). It is unknown if glucose transporters were upregulated by these insulin increases. Traumatic brain injury, known to result in an 8-fold increase in extracellular lactate (Nilsson et al., 1990), led to an increase in neural (GLUT3), but not glial (GLUT1) glucose transporters (Hamlin et al., 2001). This would be consistent with increased need for energy in the neural compartment.

### Glycogen

Astrocytes are the primary storage location for glycogen (Cataldo and Broadwell, 1986). Glycogen is broken down to glucose, but studies have shown that glycogen mobilization in astrocytes provides lactate, not glucose, to the extracellular milieu (Dringen et al., 1993). During periods of aglycemia, astrocytic glycogen is rapidly depleted, able to sustain function for no more than a few minutes (Brown, 2004). Increasing glycogen stores, though, can maintain axon firing longer during periods of hypoglycemia in rat brain (Suh et al., 2007). Astrocytes form the glia lamina of the optic nerve head in rodents (Figure 6), and these cells undergo profound morphological change with ocular hypertension (Sun et al., 2009; Dai et al., 2012; Bosco et al., 2016; Cooper et al., 2016). Of note, the optic nerve head glia impacted by glaucoma no longer contact the basal lamina of the nerve (Dai et al., 2012), the ultrastructural location of glycogen granules (Brown, 2004). This suggests astrocyte glycogen might be a source of metabolic vulnerability in the glaucomatous optic nerve due to possible depletion. Increasing glycogen stores, however, may not make a significant difference to energy homeostasis in a chronic disease like glaucoma because of its rapid depletion. Glycogen has other potential roles though, such as support of glutamatergic neurotransmission (Obel et al., 2012). Glycogen production through glycogen synthase is regulated by three kinases, including glycogen synthase kinase 3 (GSK-3). GSK-3 in particular has probable implications for glaucoma through its targets and its promiscuity; GSK-3 has more predicted substrates than any other kinase (Linding et al., 2007). GSK-3 phosphorylation regulates transcription factors such as NF-κB, STAT3, Fos/Jun AP-1, p53 (Beurel et al., 2015); histone deacetylases; and proteins like the microtubule binding tau (Mandelkow et al., 1992), critical to Alzheimer's disease and neurodegenerative disease pathology (Stamer et al., 2002; Mandelkow et al., 2003). It is as yet unknown whether a connection exists between astrocyte glycogen production and neurodegenerative disease pathology through GSK-3.

### Creatine

Ischemia is also postulated to occur as a result of poor blood flow at the optic nerve head, as observed in glaucoma patients (Schwartz, 1994; Grunwald et al., 1998); it also occurs in models of acute IOP increase (Sun et al., 2010). ATP turnover in cells is fast, but more so in tissues subjected to ischemia. ATP supply is maintained by a pool of phosphocreatine that exists at sites of high energy consumption because phosphocreatine can be converted by creatine kinase into ATP + creatine more quickly than ATP can be generated. Creatine is neuroprotective in many models of hypoxia, including for cerebral ischemia when delivered intercerebroventricularly (Lensman et al., 2006), and after experimental stroke when the creatine is in a formulation that extends half-life (Perasso et al., 2009). Pre-treating cortical axons with creatine prevented ischemia-induced damage as well as alleviating ATP depletion (Shen and Goldberg, 2012). This protection was independent of glial cells. Nevertheless, protection from ischemia corroborated previous studies demonstrating that creatine is not effective when delivered post-ischemia injury (Lensman et al., 2006; Shen and Goldberg, 2012). It may be the case that sufficient levels of creatine could not be delivered in a timely manner to counteract the depolarization and subsequent energy depletion in ischemia.

### NMNATs

Similar to studies in sensory neurons, NMNATs have significant impact on RGCs and their axons. Embryonic mice (E18.5) nullizygous for NMNAT2 showed truncated RGC axons; the axons did not reach the optic chiasm, and there was no optic tract. RGC bodies appeared normal, with proximal axons that formed the optic nerve (Gilley et al., 2013). These results suggested a developmental role for NMNAT2 in axon extension. NMNAT3 upregulation in two different mouse models of glaucoma exerted significant protection of axons that could be reversed with treatment by 3-methyladenine, a PI3K inhibitor that reduces autophagy (Kitaoka et al., 2013). Cytoplasmic NMNAT1 upregulation protected RGC somas and axons in models of retinal ischemia and ocular hypertension (Zhu et al., 2013). The studies did not thoroughly examine the mechanism of NMNAT protection, though the apparent connection to autophagy in the Kitaoka study suggests that recycling of all but the most basic cellular components can help maintain energy homeostasis in RGCs subjected to pressure-induced insult.

### NAD+-sirtuin-PGC-1α axis manipulation

NAD+ levels decrease with aging (Guarente, 2014), and various studies of NAD+ replenishment (either direct or through precursors) demonstrated better metabolic health and mitochondrial function in diet-related diabetes (Yoshino et al., 2011), and in muscle stem cells in aged mice (Zhang et al., 2016). After axotomy *in vitro*, exogenous application of NAD+ was sufficient to slow the degeneration (Wang et al., 2005). Thus, a successful strategy for slowing or preventing axon degeneration has been to indirectly provide energy (Araki et al., 2004; Chung et al., 2013). Wallerian degeneration, the most likely mechanism by which glaucomatous RGC axons die, can be halted through increased NAD+ biosynthesis (Araki et al., 2004) and the action of a serine protease inhibitor that prevents ATP decrease (Ikegami et al., 2004). Despite NAD+ being an obvious candidate effector for axon protection, overexpression of Wld^*s*^ and NMNAT prevents axon degeneration but does not increase basal NAD+ levels (Mack et al., 2001; Araki et al., 2004), and NAD+ levels are not essential to axon preservation (Sasaki et al., 2009). If NAD+ levels are dispensable, then perhaps it is ATP levels that are crucial to axon protection. Reducing axon ATP causes irreversible axon damage (Shen et al., 2013).

NMNAT overexpression has had some success in models of glaucoma (Kitaoka et al., 2013), though if energy support is the goal, a very promising candidate is the NAD+ precursor nicotinamide riboside (Figure 4). Nicotinamide riboside (NR), when orally delivered to humans and mice, exhibits superior bioavailability and pharmacokinetics than either nicotinamide or nicotinic acid. NR significantly increases NMN, NAM, and NAD+ levels in humans by the fourth dose of a daily 1,000 mg intake, and maintains those increases up to 24 h after the seventh dose (Trammell et al., 2016).

### Sirtuins

The sirtuins, class III histone deacetylases (HDACs), have had variable impact on degeneration in the CNS, their activation effective at delaying neurodegeneration in some situations, but not all (Conforti et al., 2007). Indirect maintenance of NAD+ pools through HDAC *inhibition* has been demonstrated in neurodegenerative diseases (Langley et al., 2005). In a transgenic mouse model of Alzheimer's disease, nicotinamide treatment inhibited SIRT1 activity, reduced phosphorylated tau, and restored cognitive ability (Green et al., 2008). As was discussed above, PARP1 and SIRT1 are enzymes that require NAD+ for activity. PARP1 inhibition or elimination did not improve axon regeneration after optic nerve transection in mice (Wang et al., 2016), though treatment started 3 days after injury and there is only a few hours between injury and commencement of Wallerian degeneration.

Resveratrol, a SIRT1 activator, has been utilized as a tool to probe the complexities of SIRT1 and degeneration. Resveratrol delayed Wallerian degeneration in sensory neuron cultures and after sciatic nerve crush (Calliari et al., 2013). The degeneration delay could be blocked by eliminating an endogenous SIRT1 inhibitor, DBC-1. DBC-1 knockout nerves degenerated just as quickly as wildtype if NAD+ was not present, indicating that NAD+ was necessary for full SIRT1 activity and subsequent axon protection (Calliari et al., 2013). Resveratrol also induced phosphorylation of AMPK, a master regulator of energy homeostasis (Dasgupta and Milbrandt, 2007), which when activated, stimulates glucose uptake in the brain (Hardie et al., 2012).

Resveratrol increased mitochondrial number and improved mitochondrial function primarily through SIRT1 deacetylation of PGC-1α and subsequent increased PGC-1α activity in mice (Lagouge et al., 2006). These effects were observed in skeletal muscle and adipose tissue. In the CNS, resveratrol was found to increase brain lactate production and limit movement of radiolabeled pyruvate through Krebs cycle, thereby limiting oxidative phosphorylation (Rowlands et al., 2015). Is limiting oxidative phosphorylation a viable approach for preventing axon degeneration? The variable impact (positive and negative) of SIRT1 on Wallerian degeneration indicates the likelihood that the timing and overall energy state of the system dictate the response to SIRT1 activation. Blocking oxidative phosphorylation would limit ROS production, though blocking mitochondrial function, especially if axons are being supported by glial-derived lactate, would seem to be a risky approach.

SIRT1 manipulation has not been wholly favorable in glaucoma-related models. SIRT1 overexpression promoted RGC survival after optic nerve crush in mouse, an effect that was more pronounced than resveratrol treatment (Zuo et al., 2013). SIRT1 knockout did not increase RGC death over wildtype mice after optic nerve crush, suggesting complex interactions of cell death mechanisms. Moreover, functional testing of the RGCs showed no difference among the SIRT1 activation, overexpression, or knockout groups, suggesting that axon degeneration occurred regardless of SIRT1 status (Zuo et al., 2013). The SIRT1 downregulation that resulted from 60 min of retina ischemia in mice could be reversed with once daily mangiferin treatment, a xanthonoid antioxidant (Kim et al., 2015). Resolution of the potential impact of manipulating these key regulators of energy homeostasis in glaucoma will require closer investigation in order to determine if they might be reasonable therapeutic targets.

### Mitochondria

Upregulating mitochondrial biogenesis would provide more energy-producing organelles to susceptible RGCs, but may place stressors on protein synthesis machinery, especially with aging. Greater numbers of mitochondria would still need to get trafficked along the axon, which may pose a significant problem depending on the stage of glaucoma. One regulatory complex important for mitochondrial biogenesis, mTOR, was no different in tissues between patients with ocular hypertension (OHT) and those with glaucoma (Lascaratos et al., 2015) even though the OHT patients demonstrated increased mitochondrial efficiency and no optic neuropathy. No change in mTOR suggests that the advantage conferred to OHT patients resistant to optic neuropathy did not include a drive toward making more mitochondria. Rather than make more mitochondria, a better strategy could be to improve mitochondrial function. Compensation for impaired oxidative phosphorylation in OPA1-linked autosomal dominant optic atrophy (ADOA) patients has been associated with preservation of vision (van Bergen et al., 2011). Complex II and III activity was higher in ADOA patients with better vision, and these patients had higher respiration rate. Improving mitochondrial function has been the asserted outcome for many of the abovementioned axon protection strategies such as insulin-associated PI3K/Akt activation, SIRT1 activation, and antioxidant treatment. The variable outcomes with these treatments suggest more specific changes as opposed to generalized improved mitochondrial function are necessary.

### Mitochondrial biogenesis

If unhealthy mitochondria contribute to glaucoma pathogenesis, ridding RGCs of the ROS-producing organelles would have to be coupled with mitochondrial biogenesis. A number of other neurodegenerative disease models have manipulated mitochondrial recycling or production to neuroprotective effect. Increased DNA damage response through hyperactivation of PARP-1 [poly (ADP-ribose) polymerase-1] can inhibit mitophagy by disrupting the NAD+-sirtuin-PGC-1α axis in cells developed from xeroderma pigmentosum group A patients (Fang et al., 2014b). Mitophagy in this context can be rescued by upregulating mitochondrial uncoupling protein 2 (UCP2) or providing the NAD+ precursors nicotinamide riboside (NR) or nicotinamide mononucleotide (NMN). Resumption of mitophagy reduced mitochondrial mass, but also reactive oxygen species in cultured hippocampal neurons subjected to H_2_O_2_ or oxygen glucose deprivation (Fang et al., 2014a). Upregulation of UCP2, a target of PGC-1α, increases ATP levels, reduces ROS and can protect neurons from mitochondrial Complex I disruption in a *Drosophila* model of Parkinson's disease (Islam et al., 2012). However, UCP2 overexpression in the G93A SOD1 mouse model of ALS led to lower levels of ROS but acceleration of the disease course (Peixoto et al., 2013). The proton conductance function of UCP2 requires the presence of specific activators that include fatty acids and ROS-derived alkenals (Brand and Esteves, 2005), the lack of which might explain why UCP2 overexpression is not always protective. Necdin, a protein that binds SIRT1, can stabilize PGC-1α and inhibit its ubiquitin-dependent degradation, thereby enhancing mitochondrial biogenesis (Hasegawa et al., 2016). The neuroprotection against mitochondrial-related injury afforded by necdin resembles that shown by PGC-1α upregulation (Mudò et al., 2012).

### Ketone bodies

Insight into the potential role of metabolism in the pathogenesis of glaucoma has emerged from several recent studies. In the EAAC1−/− model of normal tension glaucoma, every-other-day-fasting (EODF) led to significant protection of FluoroGold-positive retrogradely labeled RGCs, as well as the b wave of multi-focal ERG (Guo et al., 2016). EODF appeared to exert its effects through elevated β-hydroxybutyrate (β-HB), a ketone and endogenous histone deacetylase (HDAC) inhibitor. Increased β-HB levels led to increased histone acetylation that occurred concomitantly with increased mRNA expression of BDNF, bFGF, and catalase (Guo et al., 2016). β-HB can inhibit the class I HDACs (HDAC1, 2, 3, and 8) and at least one class II HDAC (HDAC4) (Shimazu et al., 2013). Ketogenic or medium chain fatty acid diets that lead to high levels of β-HB have long been effective at controlling seizure in children with epilepsy (Yellen, 2008), and have also demonstrated neuroprotection in models of Parkinson's disease (Yang and Cheng, 2010), Alzheimer's disease (Henderson et al., 2009), and ALS (Zhao et al., 2006). An important question is whether increasing ketone levels and subsequent manipulation of energy pathways is responsible for the observed neuroprotection, or if the results can be ascribed particularly to the inhibition of histone acetylation. Inhibiting HDACs has been mostly effective at limiting damage in models of glaucoma. For example, inhibiting HDAC3 protected RGCs from death after optic nerve crush (Schmitt et al., 2014), and HDAC inhibition with valproic acid protected RGCs from death and preserved pERG amplitude in a rat model of ocular hypertension (Alsarraf et al., 2014). A broad HDAC inhibitor, trichostatin A, when delivered to D2 mice weekly for 4 months, did not protect the optic nerve from degeneration (Pelzel et al., 2012). What of the possibility that β-HB limits damage because it is a ketone body? Ketone bodies get converted into acetyl-CoA for utilization in the Krebs cycle (Figure 3), the biosynthetic hub whose intermediates provide the building blocks for fatty acids, nucleotides and the carbons for gluconeogenesis. In the near term, then, increased levels of monocarboxylates like ketone bodies would require the Krebs cycle and oxidative phosphorylation for energy generation. In mammals, more lactate could feed the production of glucose through gluconeogenesis, though this is an energy-expending process. L-lactate can protect cortical neurons from excitotoxic cell death *in vitro* (Llorente-Folch et al., 2016); this effect depends upon the aspartate-glutamate carrier in CNS mitochondria, ARALAR/AGC. This carrier is one half of the malate-aspartate shuttle that moves malate/aspartate and α-ketoglutarate/glutamate between the cytoplasm and the mitochondria in order to regenerate NAD+ from NADH in both compartments. Neuronal utilization of L-lactate depends on the this pathway (Llorente-Folch et al., 2016), underscoring the importance of NAD+ availability to managing the effects of too much NMDA receptor activation. This is one potential mechanism of ketone body neural protection that is separate from HDAC inhibition.

## Resolving metabolic vulnerability

Aging is a primary risk factor in glaucoma. There is recognition across neurodegenerative diseases that glucoregulatory control decreases with aging, spawning clinical trials designed to ameliorate the effects of aging through the use of compounds like metformin (ClinicalTrials.gov Identifier: NCT02432287) or acarbose (Brewer et al., 2016). These trials do not address glaucoma in particular, but may impact the disease through improved glucoregulatory control. Serum citrate as a potential biomarker for glaucoma was the subject of a recently completed clinical trial that showed significantly decreased citrate in the serum of Caucasian glaucoma patients (Fraenkl et al., 2011). Citrate is a byproduct of the Krebs cycle, so decreased values support the idea that mitochondrial function is impaired in glaucoma patients. Mitochondrial function can be monitored in patient retinas using the green fluorescence emitted by oxidized flavoproteins, a byproduct of oxidative stress (Field et al., 2011). This development may improve diagnosis as well as provide a means to monitor future metabolic therapy.

The complexity of optic nerve degeneration in glaucoma—its chronic timing, its asynchronicity, its non-cell autonomous nature—presents many challenges to medicine. Having reviewed the various ways energy plays a role in axon degeneration, we have also summarized several metabolic-related mechanisms of protection. The evidence indicates energy plays a critical role in the timing and organization of axon degeneration, including in glaucoma.

### Note added in proof

Significant RGC soma and axon protection from glaucoma-related degeneration in the D2 mouse was achieved through dietary supplementation of nicotinamide (Williams et al., 2017). The finding is quite robust, with significant protection at both low (550 mg/kg/day) and high levels (2,000 mg/kg/day) of nicotinamide delivery. The study used RNA-sequencing to show a reversion of gene expression to that demonstrated by the control strain, DBA/2-Gpnmb^+^, across clusters of genes that included those involved in oxidative phosphorylation; reactive oxygen species, glucose, and fatty acid metabolism; and DNA repair. These results support the existence of a metabolic vulnerability in glaucoma; the mechanism of protection is yet to be elucidated.

## Author contributions

DI conceived of the topic and outlined the review; DI and MH wrote the review.

## Funding

This work was supported by NIH EY026662 (DI).

### Conflict of interest statement

The authors declare that the research was conducted in the absence of any commercial or financial relationships that could be construed as a potential conflict of interest.
